# Multi-omics analysis identifies macrophage immunometabolic signatures in ulcerative colitis

**DOI:** 10.3389/fcimb.2026.1858403

**Published:** 2026-09-03

**Authors:** Rui Zhang, Huifang Zhang, Zhifeng Wang, Yonggang Wang, Zhongwei Jia

**Affiliations:** 1 Department of Colorectal Surgery, Fifth Hospital of Shanxi Medical University, Shanxi Provincial People’s Hospital, Taiyuan, China; 2 Department of Occupational Health, School of Public Health, MOE Key Laboratory of Coal Environmental Pathogenicity and Prevention, Shanxi Medical University, Taiyuan, China; 3 Department of Gastroenterology, Fifth Hospital of Shanxi Medical University, Shanxi Provincial People’s Hospital, Taiyuan, China; 4 Fifth Hospital of Shanxi Medical University, Shanxi Provincial People’s Hospital, Taiyuan, China

**Keywords:** machine learning, macrophages, metabolic reprogramming, ScRNA-seq, ulcerative colitis, WGCNA

## Abstract

**Background:**

Ulcerative colitis (UC) is a chronic inflammatory bowel disease characterized by intestinal immune dysregulation and mucosal barrier dysfunction. Macrophages play central roles in gut immunity, yet their metabolic reprogramming and heterogeneity in UC remain insufficiently characterized.

**Methods:**

We integrated single-cell RNA sequencing (scRNA-seq), bulk transcriptomics, weighted gene co-expression network analysis (WGCNA), and machine learning algorithms (LASSO, SVM-RFE, and Random Forest) to identify candidate macrophage-associated metabolic regulators in UC. Pseudotime trajectory, CellChat, and functional enrichment analyses were used to assess differentiation, intercellular interactions, and pathway involvement. Diagnostic performance was evaluated using ROC curves and validated in independent datasets. Experimental validation was performed using Hematoxylin and Eosin (H&E) staining for histopathological assessment and Western blotting for protein expression analysis.

**Results:**

scRNA-seq identified 505 macrophage-specific genes, with pseudotime analysis suggesting differentiation branches marked by RGCC and FOSL2. Transcriptomic profiling revealed 1,058 differentially expressed genes, enriched in TNF, epithelial–mesenchymal transition, and NF-κB pathways. Six macrophage-associated immunometabolic genes (CYBB, CR1, INPP5D, CTSH, IFI16, and NCF4) were identified through WGCNA and machine learning analyses. These genes were strongly correlated with macrophage infiltration and cytokine signaling, showing high diagnostic performance (AUC > 0.98), although potential overfitting cannot be fully excluded. Consensus clustering stratified UC patients into two molecular subtypes, with Cluster 1 exhibiting a proinflammatory phenotype. H&E staining confirmed characteristic mucosal inflammation and epithelial damage in UC tissues, while Western blotting validated the upregulation of the six key regulators in UC tissues.

**Conclusion:**

This multi-omics analysis identifies six macrophage metabolic regulators (CYBB, CR1, INPP5D, CTSH, IFI16, and NCF4) with potential diagnostic relevance in UC. Experimental validation provides supportive evidence for their association with macrophage-related immunometabolic alterations in UC.

## 1 Introduction

Ulcerative colitis (UC), a major subtype of inflammatory bowel disease (IBD), is a chronic and relapsing inflammatory disorder of the colon that imposes a substantial global health burden. Historically, its incidence was highest in North America and Europe, reaching 10–20 cases per 100,000 individuals annually (Ungaro et al., 2017). More recently, a rapid rise has been reported in Asia, particularly in China, where the incidence in urban areas now approaches 8.2 per 100,000 population (Zhao et al., 2013; Ananthakrishnan et al., 2020). This epidemiological shift has been linked to lifestyle changes, urbanization, and alterations in the gut microbiota, underscoring UC as an urgent public health challenge that demands improved mechanistic insights and therapeutic strategies.

UC is characterized by persistent mucosal inflammation, abdominal pain, diarrhea, and hematochezia, which severely impair quality of life (Zhou et al., 2023; Caron et al., 2024). Its pathogenesis involves a complex interplay between genetic predisposition, environmental triggers, and dysregulated immune responses (Ananthakrishnan et al., 2020). While bulk RNA-seq studies have revealed transcriptomic changes in UC tissues (Arijs et al., 2009), they cannot fully resolve cellular heterogeneity. Single-cell RNA sequencing (scRNA-seq) now enables high-resolution characterization of immune cell diversity and differentiation in UC (Smillie et al., 2019). Nevertheless, cell states across different disease stages remain incompletely characterized, multi-omics integration is still limited, and systematic validation of candidate marker genes is still lacking (Parikh et al., 2019; Chen et al., 2024).

Among innate immune cells, macrophages are particularly abundant in the intestinal lamina propria and play central roles in pathogen clearance, antigen presentation, and cytokine production (Han et al., 2021). Their plasticity allows dynamic adaptation to environmental cues, but in UC, dysregulated macrophage activation sustains chronic inflammation. An imbalance between pro-inflammatory M1 and anti-inflammatory M2 macrophages, together with extensive crosstalk with T cells, B cells, and other immune subsets, amplifies the inflammatory cascade and may contribute to disease progression (Na et al., 2019; Zhang et al., 2023). Despite their importance, the genetic and molecular mechanisms connecting macrophage heterogeneity with UC progression remain poorly defined.

Recent studies highlight immunometabolic reprogramming as a critical determinant of macrophage polarization and function (Kelly and O'Neill, 2015; Liu et al., 2021). Shifts in glucose, fatty acid, and amino acid metabolism allow macrophages to adopt distinct inflammatory or regulatory phenotypes, thereby influencing immune homeostasis (Viola et al., 2019; Ryan and O'Neill, 2020). While this paradigm has been extensively studied in cancer and infectious diseases, its contribution to UC and its genetic basis remain largely unexplored. Whether metabolic reprogramming underlies macrophage heterogeneity in UC, and how it influences disease subtypes, are unresolved questions.

Given these significant gaps in our understanding, this study aims to investigate the role of macrophage immunometabolic reprogramming in UC. By integrating single-cell RNA sequencing (scRNA-seq) and bulk transcriptomics, we sought to identify macrophage-associated immunometabolic signatures linked to macrophage heterogeneity and metabolic alterations in UC. Targeted validation using molecular biology techniques provides preliminary support for their involvement in disease progression. These findings not only provide novel insights into macrophage-driven immunometabolic dysfunction but also offer potential biomarkers for precision stratification in UC. Our study contributes to the growing body of research linking metabolic reprogramming with immune dysfunction in chronic inflammation, and provides a systematic approach to uncovering therapeutic targets in UC.

## 2 Methods

### 2.1 Data acquisition and preprocessing

A total of 220 samples were obtained from five datasets (GSE162335, GSE48958, GSE73661, GSE75214, and GSE16879) in the Gene Expression Omnibus (GEO) database (https://www.ncbi.nlm.nih.gov/geo/) (**Supplementary Table 1**) (Barrett et al., 2005). For the scRNA-seq dataset GSE162335, stringent quality control was applied by removing cells with >5% mitochondrial gene content and those with extremely low (≤500) or high (≥6000) detected gene counts, resulting in 17,936 cells and 19,402 genes. Importantly, GSE162335 contains only UC-derived samples and does not include healthy colonic controls. Therefore, the scRNA-seq analysis was used to characterize macrophage-associated transcriptional programs and cellular heterogeneity within UC rather than to identify disease-specific macrophage alterations relative to healthy tissue. For bulk RNA-seq datasets (GSE48958, GSE73661, GSE75214, and GSE16879), CEL files and platform annotation files were downloaded and normalized using the affy package (Gautier et al., 2004). Probe IDs were converted into gene symbols, discarding unmatched probes and averaging values when multiple probes mapped to the same gene. Additionally, 256 metabolism-related pathways were retrieved from the MSigDB database (**Supplementary Table 2**) (Liberzon et al., 2011) to support downstream analyses.

### 2.2 Identification of macrophage signature genes via scRNA-seq

scRNA-seq data were normalized using Seurat, and 3,000 highly variable genes were identified with the *FindVariableFeatures* function. Principal component analysis (PCA) was applied to select relevant components for clustering, which was performed with *FindNeighbors*, *FindClusters* (resolution = 0.5), and *RunUMAP*. Marker genes for each cluster were annotated using PanglaoDB and CellMarker 2.0 (Franzén et al., 2019; Zhang et al., 2019). Macrophage differentiation trajectories were reconstructed with Monocle (Trapnell et al., 2014), while ligand–receptor interactions among subpopulations were inferred using CellChat (Jin et al., 2021). Enrichment of macrophage marker genes was assessed by Gene Ontology (GO) and Kyoto Encyclopedia of Genes and Genomes (KEGG) analyses implemented in clusterProfiler (Yu et al., 2012).

### 2.3 Differential expression and functional enrichment in UC RNA-seq analysis

Batch effects across datasets were assessed via PCA and corrected using the SVA package (Leek et al., 2012). Differential expression between UC and control samples was determined using the limma package (Albaradei et al., 2021), applying a Bayesian linear regression framework. Genes with adjusted *P* < 0.05 and |log2FC| > 1 were considered significant. Gene Set Enrichment Analysis (GSEA) (Subramanian et al., 2005) was used to identify functional differences, with hallmark enrichment performed using MSigDB gene sets. Significance thresholds were set at |NES| > 1, adjusted *P* < 0.05, and gene count ≥5.

### 2.4 Macrophage-related module gene analysis via RNA-seq, EPIC, ssGSEA, and TIMER

Immune infiltration analysis was conducted using four algorithms, CIBERSORT (Chen et al., 2018), EPIC (Racle and Gfeller, 2020), ssGSEA (Xiao et al., 2020), and TIMER (Li et al., 2017). Infiltration differences between UC and control groups were tested with the Wilcoxon rank-sum test and visualized in box plots. Correlations among immune cell populations were derived from ssGSEA results. Weighted gene co-expression network analysis (WGCNA) (Langfelder and Horvath, 2008) was used to identify macrophage-related modules. Module genes were further analyzed for functional enrichment (GO biological process, cellular component, molecular function, and KEGG pathways) with thresholds set at count ≥2 and adjusted *P* < 0.05.

### 2.5 Machine learning-based feature gene selection

Genes from macrophage-related modules were intersected with metabolism-related gene sets and scRNA-seq-derived macrophage marker genes to identify macrophage-associated metabolic signatures. Because macrophage marker genes primarily reflect cellular identity rather than regulatory function, the resulting candidates were interpreted as macrophage-associated immunometabolic genes rather than definitive metabolic regulators. Three machine learning models-LASSO-logistic regression (*glmnet*) (Simon et al., 2011), Support Vector Machine- Recursive Feature Elimination (SVM-RFE, *e1071*) (Yang et al., 2022), and Random Forest (*randomForest*) (Utkin and Konstantinov, 2022) were used to identify diagnostic feature genes. Genes consistently identified across all models were designated as final feature genes. Their expression differences between UC and control groups were validated with the Wilcoxon test. Diagnostic performance was evaluated using ROC curves generated by *pROC* (Robin et al., 2011). A nomogram was constructed, with calibration, decision, and ROC curves applied for validation.

### 2.6 GSEA, expression patterns in cell subpopulations, and clustering analysis

Spearman correlation analysis was performed to evaluate associations between feature genes and immune cell infiltration scores. UC patients were stratified into high- and low-expression groups for KEGG enrichment analysis to explore pathway alterations associated with feature gene expression. Expression patterns of feature genes were visualized across scRNA-seq subpopulations. Finally, ConsensusClusterPlus (Wilkerson and Hayes, 2010) was applied to classify UC patients into molecular subtypes, with the optimal number of clusters determined by cumulative distribution function plots and heatmaps. Subtypes were further validated by PCA visualization and differential expression analysis. To further characterize the immunological heterogeneity between subtypes, the ssGSEA algorithm was utilized to quantify the infiltration levels of 28 immune cell subsets in UC samples, and the Wilcoxon rank-sum test was used to compare differences in immune cell abundance between distinct clusters.

### 2.7 Experimental validation of key genes

To strengthen the reliability of the computational findings, both histological and protein-level validation experiments were performed on colonic tissue specimens from patients with UC and matched healthy controls. All human tissue experiments were conducted in accordance with the Declaration of Helsinki and approved by the Institutional Ethics Committee of Shanxi Provincial People’s Hospital (Approval No.: 2025-872).

### 2.8 Hematoxylin and eosin staining

Fresh colonic tissues were fixed in 4% paraformaldehyde for 24 hours, dehydrated, and embedded in paraffin. Sections of 4 μm thickness were prepared, dewaxed in xylene, rehydrated through graded ethanol solutions, and stained sequentially with hematoxylin for 5 minutes and eosin for 2 minutes. After dehydration, slides were mounted and examined under a light microscope (Olympus BX53, Tokyo, Japan). Histological assessment focused on epithelial injury, crypt distortion, inflammatory infiltration, and mucosal ulceration to confirm the classical pathological characteristics of UC.

### 2.9 Western blotting

Proteins were extracted from UC and control colonic tissues using RIPA lysis buffer supplemented with protease and phosphatase inhibitors (Beyotime, Shanghai, China). Protein concentration was determined with a BCA Protein Assay Kit (Thermo Fisher Scientific, USA). Equal amounts of total protein (30 μg per lane) were separated by SDS-PAGE and transferred onto PVDF membranes (Millipore, USA). After blocking with 5% non-fat milk in TBST for 1 h at room temperature, membranes were incubated overnight at 4°C with primary antibodies against CYBB, CR1, INPP5D, CTSH, IFI16, and NCF4 (1:1000; Abcam, UK). Subsequently, membranes were washed and incubated with HRP-conjugated secondary antibodies (1:5000; Cell Signaling Technology, USA) for 1 h at room temperature.

Owing to differences in molecular weight, β-actin served as the internal control for CR1, INPP5D, CYBB, and IFI16, whereas β-tubulin was used for NCF4 and CTSH. Bands were visualized using enhanced chemiluminescence (ECL, Bio-Rad, USA) and quantified by ImageJ software. Relative expression levels were normalized to internal controls and compared between UC and control samples using two-tailed Student’s t-tests, with *P* < 0.05 considered statistically significant.

## 3 Results

### 3.1 Identification of macrophage signature genes

**Figure 1:**
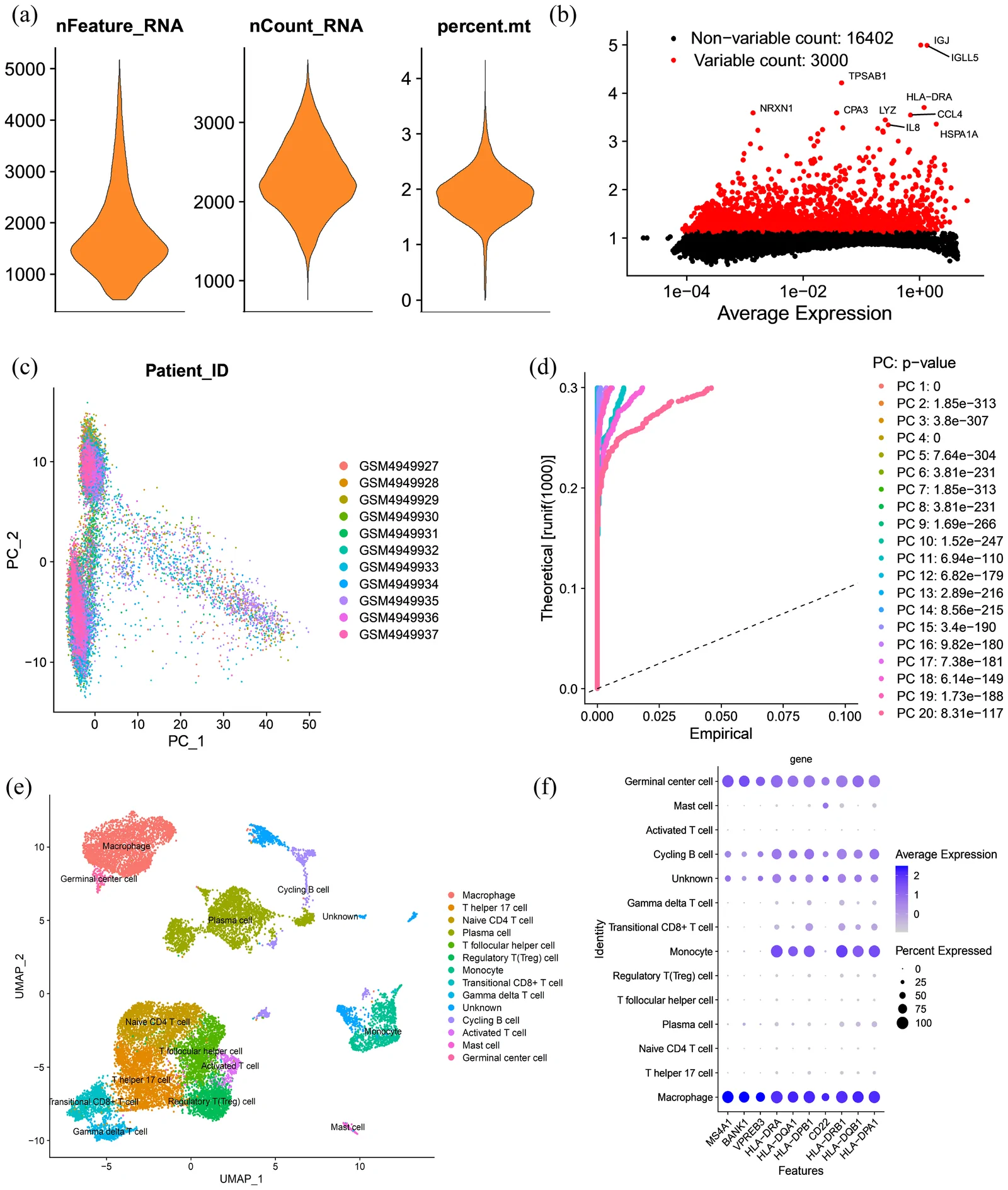
Identification of macrophage marker genes by scRNA-seq. **(a)** Data quality control of scRNA-seq profiles. **(b)** Highly variable genes (top 3,000) identified by variance plot. **(c)** PCA-based dimensionality reduction. **(d)** Selection of 20 significant principal components (P < 0.05). **(e)** Cell cluster annotation using known marker genes. **(f)** Bubble plot of the top 10 macrophage marker genes, showing expression distribution across clusters.

We analyzed scRNA-seq profiles from 17,936 cells across 11 primary UC samples (GSE162335). After stringent quality control and normalization, the top 3,000 highly variable genes were selected and subjected to PCA, which identified 20 significant principal components (PCs, *P* < 0.05) (**Figures 1a–d**). UMAP visualization resolved 20 distinct cell clusters. Cell annotation was performed using the Find All Markers function combined with Panglao DB and CellMarker 2.0 (**Supplementary Table 3**), resulting in the identification of 505 macrophage-associated genes (**Supplementary Table 4**). Representative marker genes, including MS4A1, BANK, VPREB3, HLA-DRA, and HLA-DQA1, were mapped across macrophage subclusters (**Figure 1f**). These findings provided a robust catalog of macrophage-specific signatures in UC.

### 3.2 Differentiation trajectories and functional features of macrophage subpopulations

**Figure 2:**
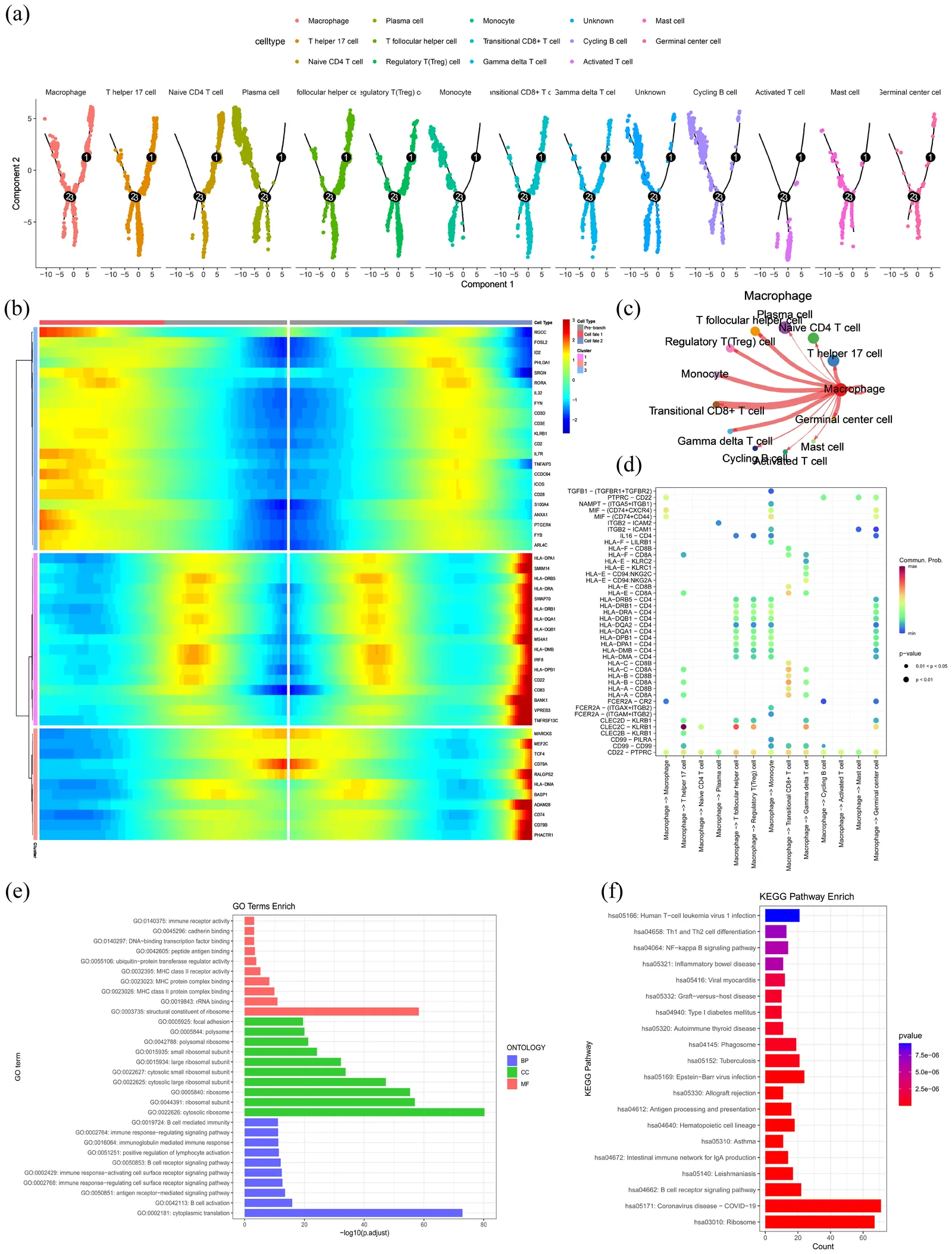
Differentiation trajectories and functional features of macrophage subpopulations. **(a)** Pseudotime trajectory analysis showing developmental branches (pre-branch, fate 1, and fate 2). **(b)** Heatmap of the top 50 dynamic genes with stage-specific patterns. **(c)** CellChat analysis of intercellular communication, highlighting macrophage signaling networks. **(d)** Selected ligand- receptor interactions illustrating macrophage–immune cell crosstalk. **(e)** GO enrichment of macrophage marker genes. **(f)** KEGG analysis highlighting enrichment in B cell receptor and NF-κB pathways.

Pseudotime trajectory analysis delineated distinct developmental branches of UC macrophages, including pre-branch, cell fate 1, and cell fate 2 (**Figure 2a**). Beam analysis generated a heatmap of the top 50 differentiation-associated genes, showing stage-specific expression patterns (**Figure 2b**). Key regulators such as RGCC (Regulator of Cell Cycle, involved in cell cycle progression and apoptosis), FOSL2 (FOS Like 2, a transcription factor driving macrophage activation and differentiation), ID2 (Inhibitor of DNA Binding 2, regulating lymphoid and myeloid differentiation), and PHLDA1 (Pleckstrin Homology-Like Domain Family A Member 1, associated with apoptosis and stress responses) were identified as strongly associated with differentiation dynamics. To further characterize immune interactions, CellChat analysis revealed extensive ligand- receptor crosstalk between macrophages and other immune cell types, particularly follicular helper T cells and monocytes (**Figures 2c, d**; **Supplementary Table 5**). GO and KEGG enrichment analyses confirmed enrichment in immune response, antigen receptor-mediated signaling, and ribosomal processes, with pathways including B-cell receptor signaling and NF-κB signaling significantly represented (**Figures 2e, f**). Together, these analyses highlight reproducible transcriptional and interaction patterns that define macrophage subpopulations in UC.

### 3.3 Differentially expressed genes in UC RNA-seq data

**Figure 3:**
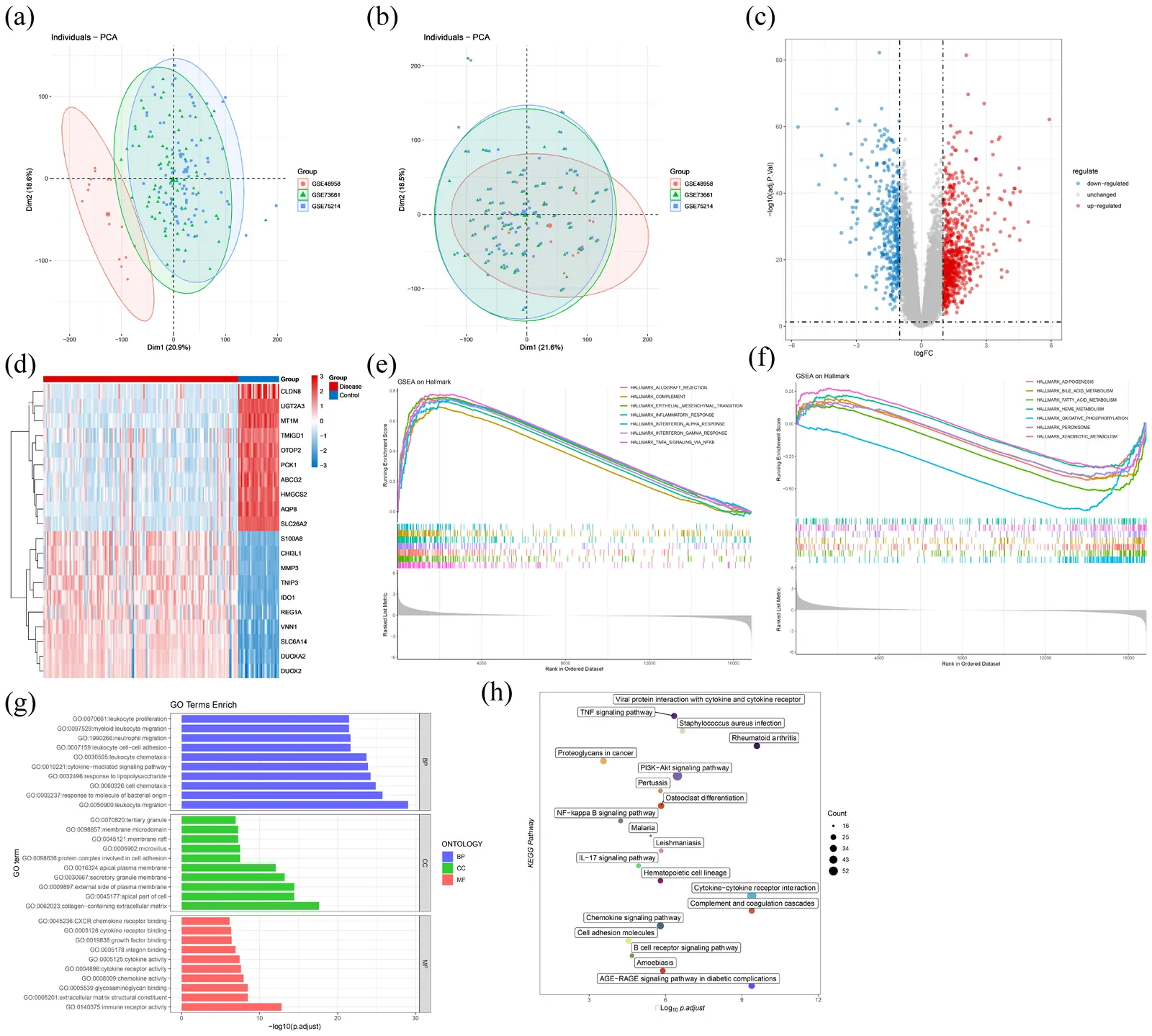
Differentially expressed genes in UC from bulk RNA-seq. **(a, b)** PCA plots before and after SVA correction of batch effects. **(c)** Volcano plot of significantly up- and downregulated genes. **(d)** Heatmap of the top 10 DEGs. **(e, f)** Hallmark enrichment showing activation of immune pathways and suppression of metabolic processes. **(g, h)** GO and KEGG analyses suggesting immune dysregulation, particularly NF-κB and PI3K-Akt signaling.

To complement scRNA-seq, we integrated three RNA-seq datasets (GSE48958, GSE73661, and GSE75214) comprising 220 samples. PCA detected batch effects, particularly in GSE48958 (**Figure 3a**), which were corrected using the SVA package (**Figure 3b**; **Supplementary Table 5**). After correction, differential expression analysis identified 1,058 DEGs between UC and controls (**Figures 3c, d**; **Supplementary Table 6**). Hallmark enrichment revealed significant upregulation of immune-related pathways such as allograft rejection, complement cascade, EMT, and TNF signaling (**Figures 3e, f**). Downregulated DEGs were enriched in fatty acid metabolism, adipogenesis, and xenobiotic metabolism, suggesting broader metabolic alterations in UC. GO terms highlighted leukocyte proliferation and chemotaxis, while KEGG pathways included NF-κB, PI3K-Akt, B cell receptor, and TNF signaling (**Figures 3g, h**). These results provide a consistent transcriptomic landscape of UC.

### 3.4 WGCNA and identification of macrophage-related gene modules

**Figure 4:**
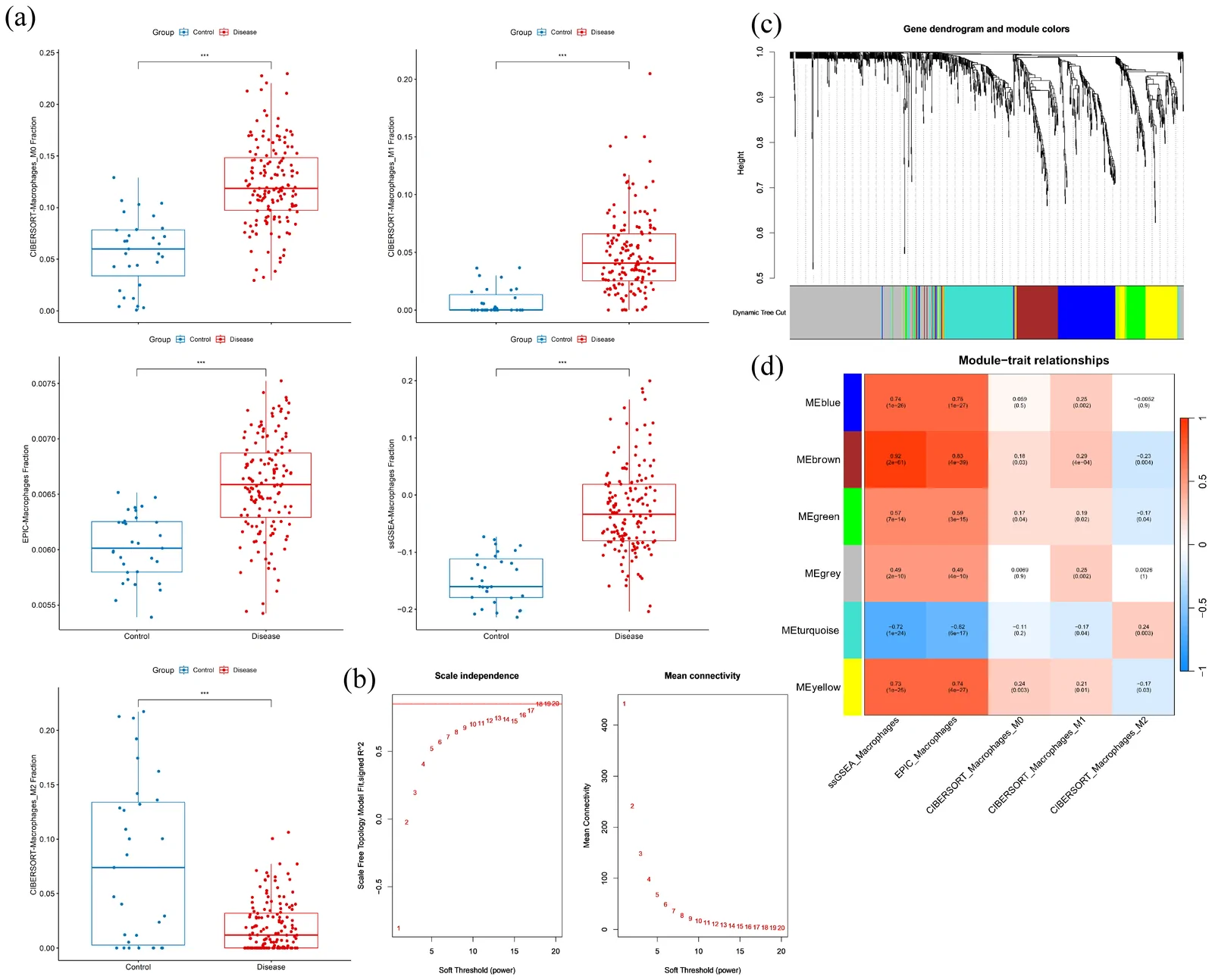
WGCNA of macrophage-related genes in UC. **(a)** Boxplot of macrophage infiltration scores across groups. **(b)** Scale-free topology analysis under different soft thresholds. **(c)** Gene clustering showing distinct co-expression modules. **(d)** Module–trait correlation heatmap, identifying significant associations with macrophage scores.

Immune infiltration scores were estimated using CIBERSORT, EPIC, and ssGSEA, ensuring robustness across methods (**Figure 4a**). Immune scores were used as the phenotypic traits to explore the association between macrophage infiltration and gene expression. WGCNA was then employed to identify co-expressed gene modules. Using a soft threshold of 18, we generated a scale-free network (**Figure 4b**), from which five modules were defined (**Figure 4c**). Three modules-blue (167 genes), brown (128 genes), and yellow (125 genes)-showed strong positive correlations (*R* > 0.70, *P* < 0.001) with macrophage infiltration scores (**Figure 4d**). In total, 420 genes were considered macrophage-related module genes (**Supplementary Table 7**). This integrative framework highlights reproducible co-expression patterns associated with macrophage activity.

### 3.5 Screening of candidate macrophage-associated immunometabolic genes in UC

**Figure 5:**
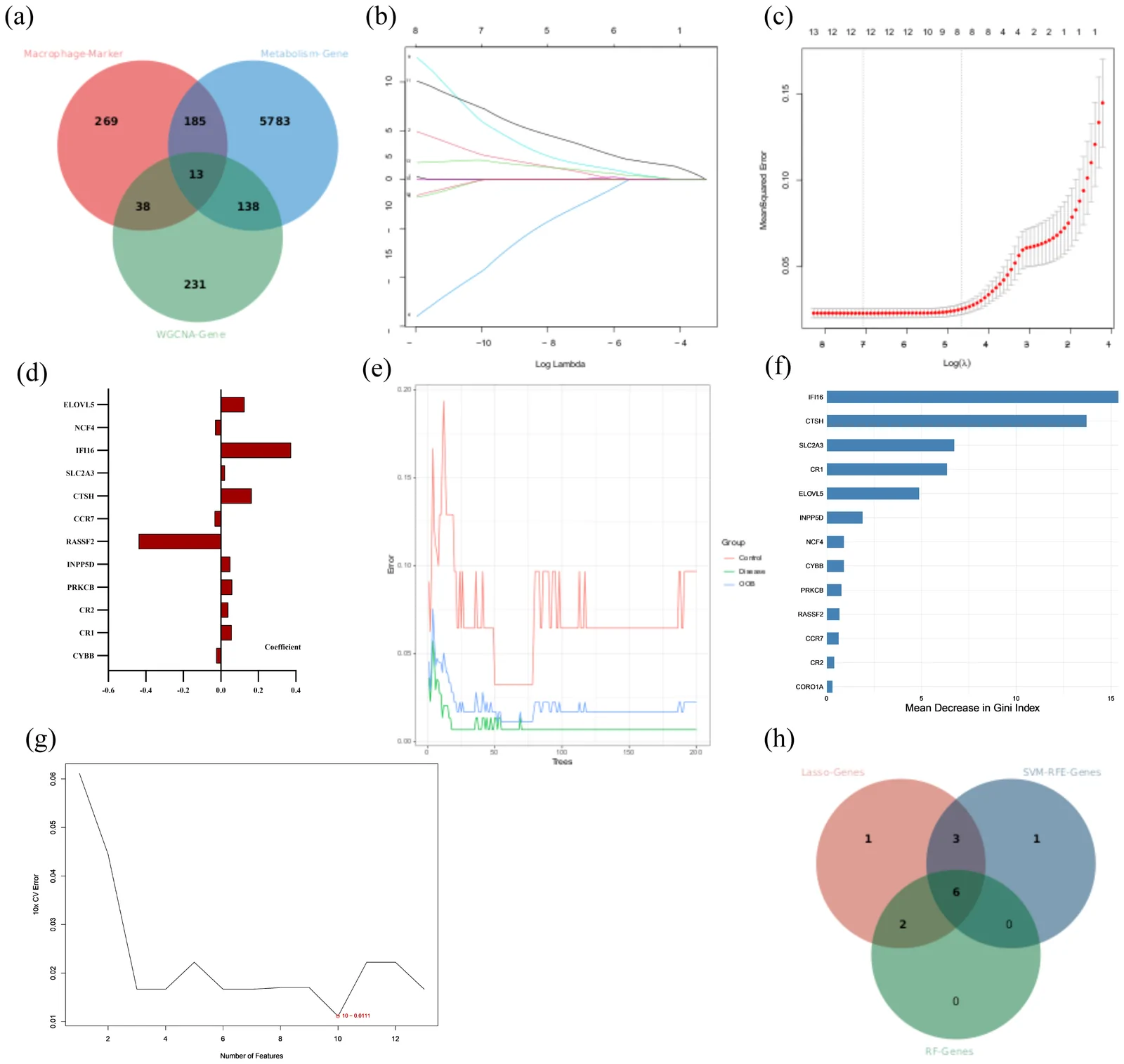
Identification of key metabolic reprogramming genes in UC macrophages. **(a)** Venn diagram of overlapping genes from WGCNA modules, metabolic reprogramming databases, and macrophage markers. **(b, d)** LASSO-logistic regression with λ-tuning and selected coefficients. **(e, f)** Random Forest ranking of gene importance. **(g)** SVM-RFE cross-validation curve for feature selection. **(h)** Venn diagram of six consistently selected genes across all machine learning methods.

To pinpoint macrophage-related metabolic regulators, we intersected WGCNA module genes, a curated metabolic gene set, and scRNA-seq macrophage markers, yielding 13 candidate genes (**Figure 5a**; **Supplementary Table 8**). To refine this list, we applied three independent machine learning algorithms:

- LASSO-logistic regression retained 12 genes (**Figures 5b–d**);
- SVM-RFE selected 10 top-ranked genes (**Figure 5g**);
- Random Forest prioritized eight genes with >95% contribution to the Gini index (**Figures 5e, f**).

The intersection yielded six consistently selected feature genes: CYBB, CR1, INPP5D, CTSH, IFI16, and NCF4 (**Figure 5h**). These reproducibly identified genes represent candidate macrophage-associated immunometabolic markers linked to macrophage abundance and inflammatory activity in UC. However, the present analyses do not establish direct regulatory or causal roles in macrophage metabolic reprogramming.

### 3.6 Diagnostic performance and clinical utility of feature genes

**Figure 6:**
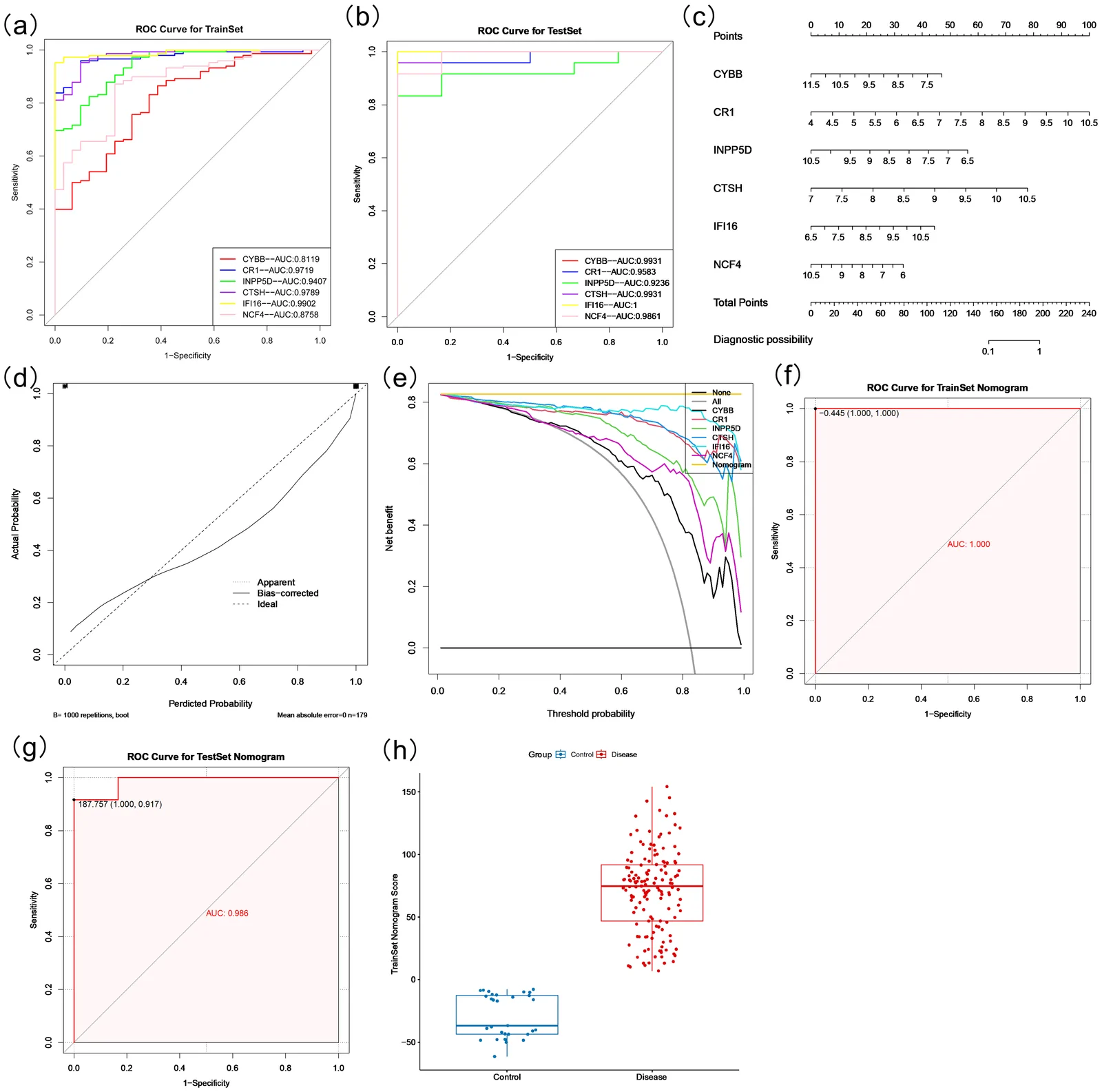
Diagnostic performance of feature genes. **(a, b)** ROC curves showing high diagnostic accuracy of six feature genes, with IFI16 and CTSH achieving AUC > 0.97. **(c)** Nomogram integrating all six genes for individualized UC risk assessment. **(d)** Calibration curve demonstrating excellent agreement between predicted and observed probabilities. **(e)** Decision curve analysis revealed superior clinical net benefit of the multigene model compared with single-gene predictors. **(f–g)** ROC curves in training and validation sets confirmed robust discrimination (AUC = 1.0 and 0.986, respectively). **(h)** Boxplot showing significantly higher diagnostic scores in UC than controls, validating clinical applicability.

The diagnostic value of the six feature genes was tested across multiple cohorts. ROC curve analysis showed AUC values of 0.81-0.99 in the training set, with IFI16 (AUC = 0.9902) and CTSH (AUC = 0.9798) performing best (**Figures 6a, b**). Validation confirmed excellent diagnostic performance (AUC 0.92-1.0), with IFI16 reaching 1.0. A nomogram incorporating all six genes provided robust predictive accuracy (**Figures 6c, d**). Decision curve analysis further demonstrated clinical utility across thresholds (**Figure 6e**). ROC validation in both training and test sets yielded AUCs of 1.0 and 0.986, respectively (**Figures 6f, g**), while box plots confirmed significantly higher diagnostic scores in UC compared to controls (**Figure 6h**). Collectively, these findings demonstrate strong discriminatory performance within the analyzed datasets. However, the exceptionally high AUC values should be interpreted cautiously because the multi-step feature-selection process and relatively limited cohort size may increase the risk of overfitting. Independent validation in larger multicenter cohorts is warranted.

### 3.7 Correlation between feature genes and macrophages

**Figure 7:**
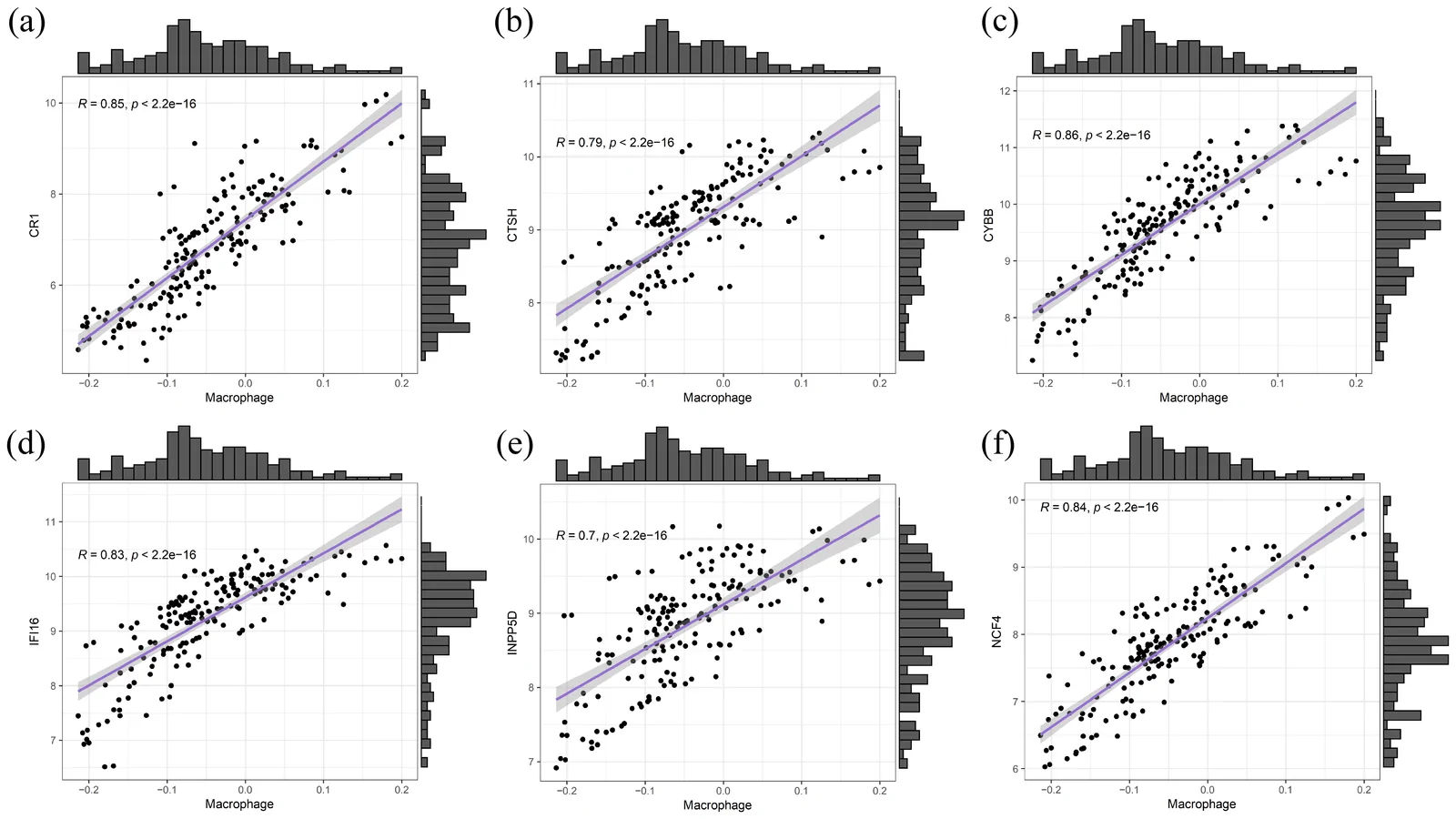
Correlation between feature genes and macrophage immune scores. Scatter plots showing correlations between the six identified metabolic reprogramming genes and macrophage immune scores. **(a)** CR1; **(b)** CTSH; **(c)** CYBB; **(d)** IFI16; **(e)** INPP5D; **(f)** NCF4. These findings highlight their potential roles in UC pathogenesis.

Spearman’s correlation analysis demonstrated significant positive associations between the six feature genes (CR1, CTSH, CYBB, IFI16, INPP5D, and NCF4) and macrophage immune scores (**Figure 7**). CYBB (*R* = 0.86, *P* < 0.05) and CR1 (*R* = 0.85, *P* < 0.05) showed the strongest correlations, followed by NCF4 (*R* = 0.84), IFI16 (*R* = 0.83), CTSH (*R* = 0.79), and INPP5D (*R* = 0.71). These results indicate that all six genes are closely linked to macrophage activity, with CYBB and CR1 emerging as central regulators. Collectively, the findings highlight their potential mechanistic roles in macrophage metabolic reprogramming and underscore their diagnostic and therapeutic potential in UC.

### 3.8 Functional roles of feature genes in UC macrophages

**Figure 8:**
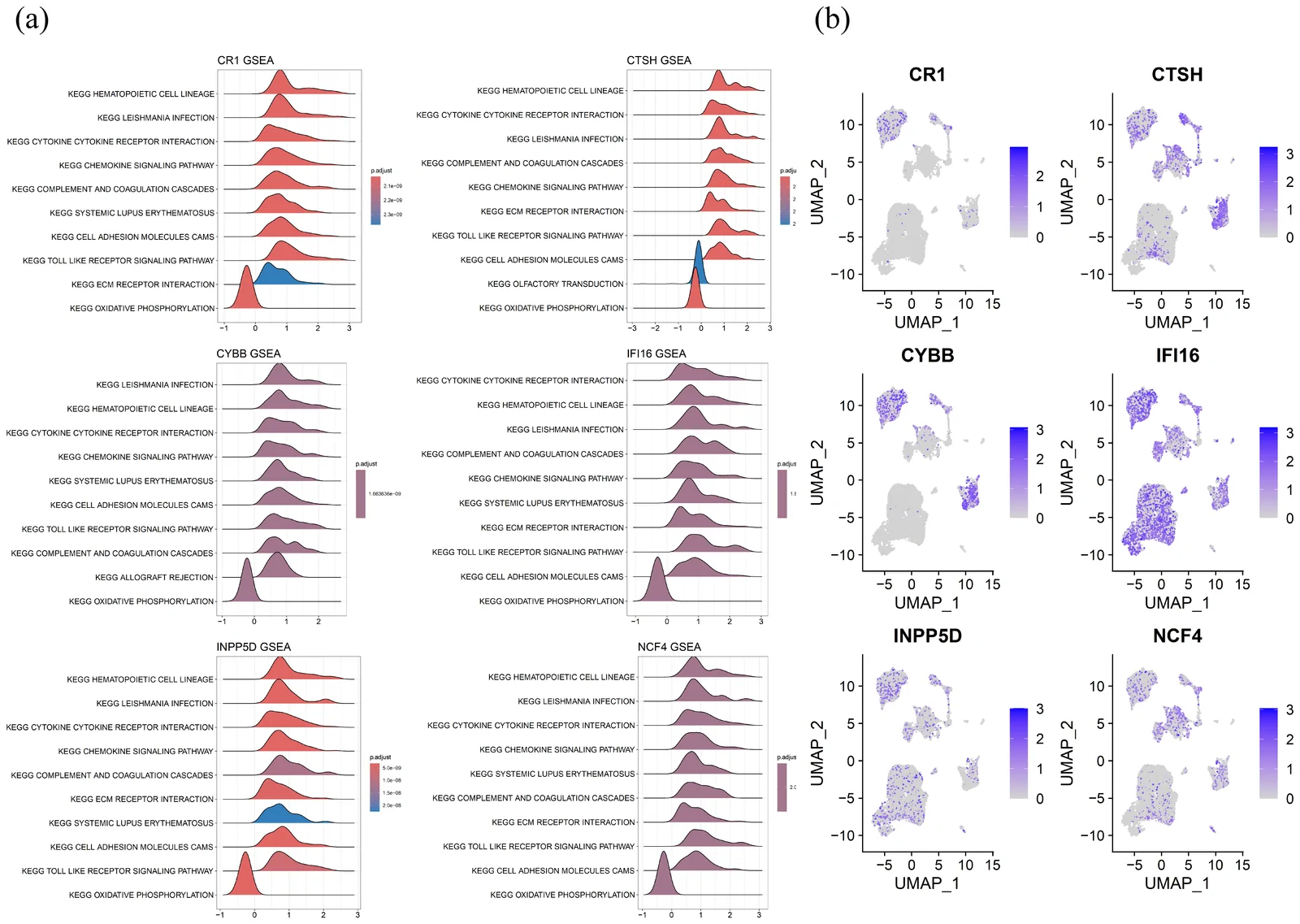
Functional enrichment and expression of feature genes. **(a)** GSEA ridge plot demonstrating enrichment of complement and coagulation cascades and cytokine–cytokine receptor pathways. **(b)** UMAP visualization showing predominant expression of CYBB and CR1 in macrophage-dominant regions, reinforcing their roles in macrophage metabolism and inflammation.

In this study, we investigated six feature genes (CR1, CTSH, CYBB, IFI16, INPP5D, and NCF4) in UC, focusing on their roles in immune regulation and macrophage metabolism (**Supplementary Tables 9**–**14**). Differential expression and GSEA enrichment analyses revealed their significant involvement in immune and inflammatory pathways, particularly complement and coagulation cascades and cytokine-cytokine receptor interactions (**Figure 8a**). UMAP clustering showed strong expression of these genes in macrophage-enriched regions, with CYBB and CR1 exhibiting especially high levels (**Figure 8b**). These findings highlight their pivotal functions in macrophage metabolic reprogramming and underscore their value as potential biomarkers and therapeutic targets for UC.

### 3.9 Molecular subtypes of UC defined by feature gene expression

**Figure 9:**
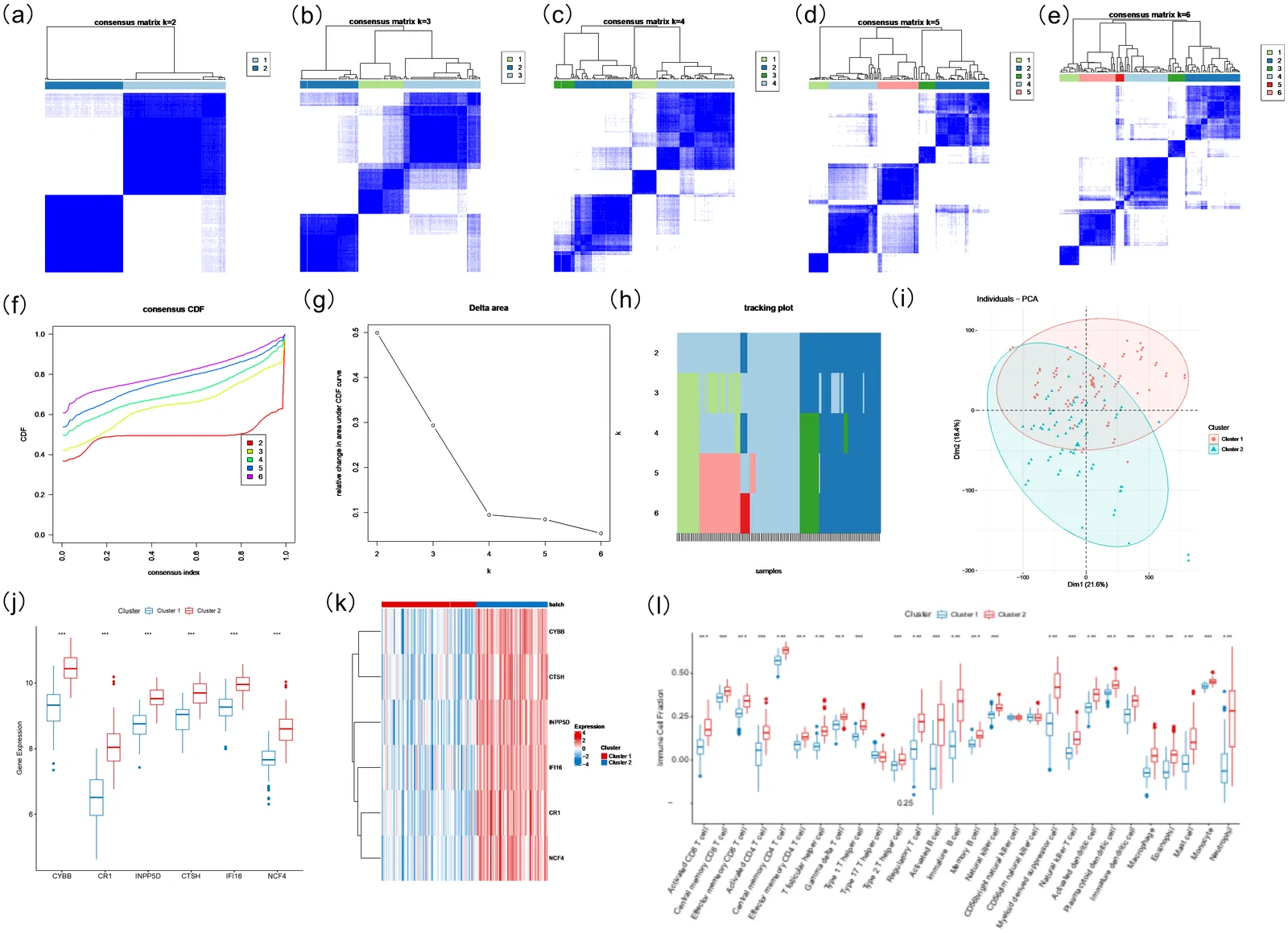
Clustering of UC patients based on feature gene expression. **(a–e)** Consensus clustering matrices dividing 148 UC patients into two stable subtypes (K = 2). **(f, g)** CDF and delta area plots validating clustering stability. **(h)** Tracking plot displaying sample assignments. **(i)** PCA plot showing clear separation of subtypes. **(j)** Boxplots indicating significantly higher expression of CYBB, CR1, and INPP5D in Cluster 1. **(k)** Heatmap visualizing expression differences, with Cluster 1 representing a metabolically active, pro-inflammatory subtype. **(l)** Box plots of 28 immune cell scores across two clusters. These results reveal metabolic reprogramming as a key mechanism underpinning UC heterogeneity and potential therapeutic stratification.

To assess clinical heterogeneity, consensus clustering of 148 UC patients identified two distinct subtypes (K = 2), comprising 84 and 64 cases (**Figures 9a–e**). PCA confirmed clear separation between subtypes (**Figure 9i**). Cluster 1 showed higher expression of CYBB, CR1, and INPP5D compared with Cluster 2 (**Figures 9j, k**), consistent with a more metabolically active and inflammatory profile. These results suggest that feature gene signatures can stratify UC patients into biologically distinct molecular subtypes. However, because detailed clinical data were unavailable for most public datasets, the clinical relevance of these subtypes requires further investigation.

To further dissect immunological discrepancies between the two subgroups, we quantified infiltration scores of 28 immune cell subsets across all UC samples via the ssGSEA algorithm, and the Wilcoxon rank-sum test was adopted to compare immune cell abundance between Cluster 1 and Cluster 2, with statistical outcomes visualized as box plots (**Figure 9l**). We found that 25 out of the 28 immune cell populations displayed markedly different infiltration levels between subtypes (all *P* < 0.001), while only CD56bright natural killer cells, CD56dim natural killer cells, and Type 17 T helper cells showed no significant inter-cluster difference. Such pervasive immune infiltration variations revealed fundamentally distinct mucosal immune microenvironment landscapes and inflammatory activation status underlying the two UC molecular subtypes.

### 3.10 Histological and protein-level validation suggests active inflammation and upregulation of macrophage-associated metabolic regulators in UC.

**Figure 10:**
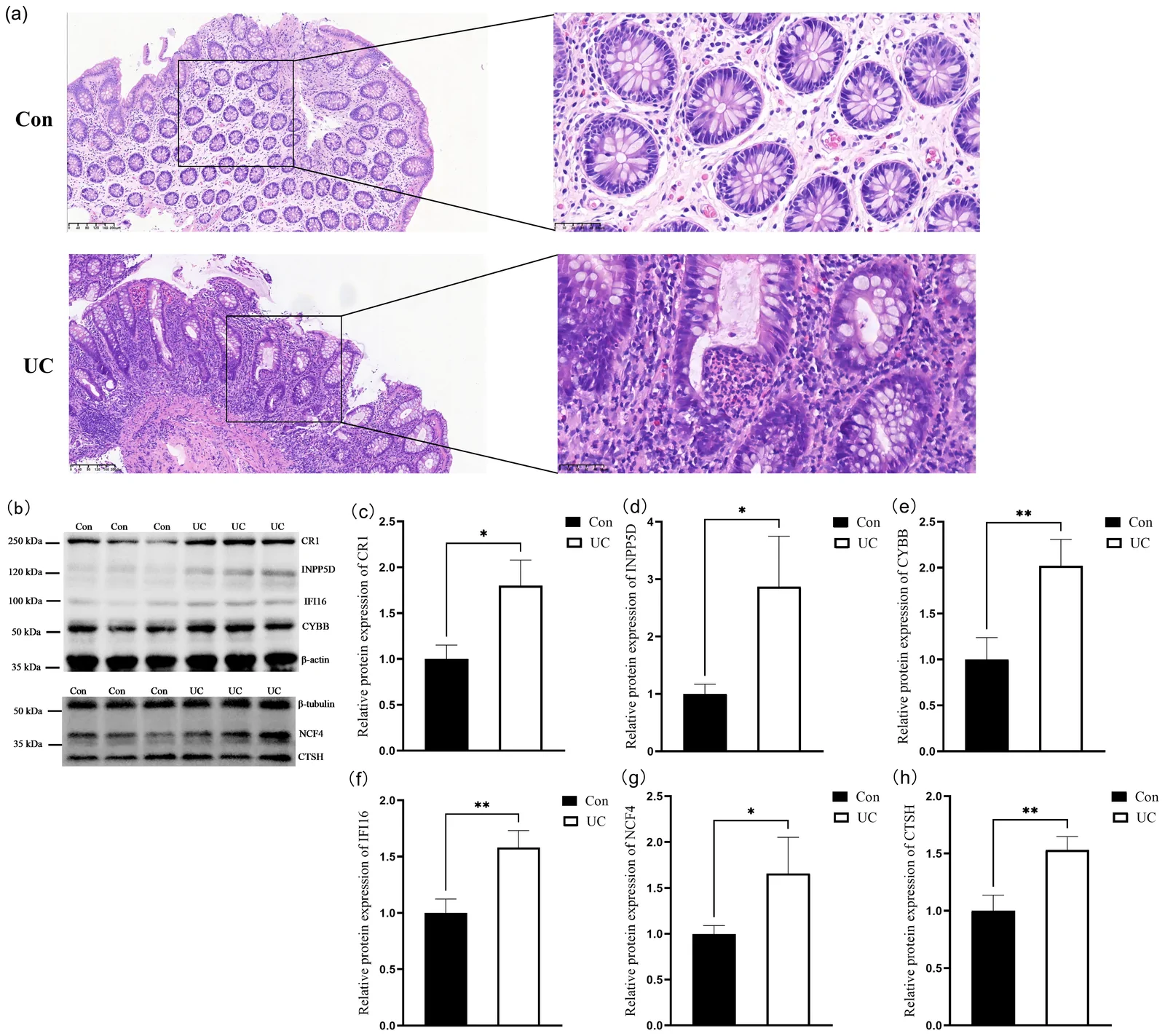
Histological and protein-level validation of mucosal inflammation and macrophage-associated gene expression in UC. **(a)** Representative H&E-stained sections of colonic tissues from UC patients and healthy controls. Scale bars: 200 μm (10×), 50 μm (40×). **(b)** Western blot analysis of six macrophage-associated metabolic regulators (CYBB, CR1, INPP5D, CTSH, IFI16, NCF4) in UC and control tissues.**(c–h)** Densitometric quantification of protein expression levels.

Histopathological examination revealed distinct mucosal injury and inflammatory infiltration in UC tissues compared with controls. H&E staining showed extensive epithelial disruption, crypt distortion, goblet cell depletion, and dense infiltration of inflammatory cells in the lamina propria (**Figure 10a**). In contrast, control tissues exhibited intact epithelial architecture with orderly crypts and minimal leukocyte infiltration. These morphological findings confirmed active mucosal inflammation and provided a histological basis for the transcriptomic evidence of innate immune activation in UC.

Western blot analysis further demonstrated significant upregulation of the six macrophage-associated metabolic regulators-CYBB, CR1, INPP5D, CTSH, IFI16, and NCF4-in UC tissues (n=3) compared with controls (n=3) (**Figure 10b**). As shown in **Figures 10 c–h**, densitometric quantification revealed the following relative increases (UC vs. control, mean ± SD): CR1,1.80 ± 0.28-fold; INPP5D, 2.87 ± 0.88-fold; CYBB, 2.02 ± 0.29-fold; IFI16, 1.58 ± 0.15- fold; NCF4, 1.66 ± 0.39-fold and CTSH, 1.53 ± 0.12-fold; (all *P* < 0.05). These results were consistent with the transcriptomic predictions and confirmed that macrophage-related metabolic pathways were significantly activated in UC mucosa.

## 4 Discussion

Macrophages are the most abundant leukocytes in the intestinal lamina propria, forming a critical immune network that preserves gut homeostasis (Lu et al., 2024; Wang et al., 2025). Dysfunctional macrophage activation is increasingly recognized as a hallmark of chronic inflammatory disorders, including IBD. Given their dual roles in inflammation resolution and tissue repair, macrophages have emerged as promising therapeutic targets across diverse pathological contexts (Na et al., 2019; Zhang et al., 2023). In this study, we generated the first single-cell differentiation atlas of UC macrophages using scRNA-seq, identifying 505 macrophage-specific genes, including MS4A1 and HLA-DRA, which are linked to immune activation in autoimmune diseases (Mattiola et al., 2021; Xiong et al., 2025). Pseudotime trajectory analysis delineated distinct differentiation branches of UC macrophages and highlighted markers such as RGCC and FOSL2, which are associated with cell cycle, apoptosis, and transcriptional regulation (Counts and Mufson, 2017; Liu et al., 2024). These results suggest that UC macrophages display unique differentiation patterns that may sustain chronic inflammation.

Beyond differentiation, CellChat analysis uncovered extensive macrophage-Tfh crosstalk, underscoring their central role in the UC immune microenvironment (Crotty, 2019). Specifically, macrophages secrete IL-6 and IL-1β and upregulate ICOSL upon inflammatory stimulation, which binds to ICOS on Tfh cells to promote their differentiation and effector functions (Ogata et al., 2013; Pyrillou et al., 2020). Conversely, Tfh cells express CD40L and secrete IL-21, which enhance macrophage activation and phagocytic activity through the CD40-CD40L and IL-21/IL-21R pathways (Liu et al., 2015). GO and KEGG enrichment confirmed that macrophage-related genes were enriched in B cell receptor and NF-κB signaling pathways, consistent with transcriptional evidence of immune activation (Papoutsopoulou et al., 2019). Together, these findings provide a comprehensive resource for exploring immune communication in UC.

Bulk RNA-seq comparison revealed over 1,000 differentially expressed genes between UC and controls. Upregulated genes were mainly enriched in the TNF signaling pathway and EMT, both recognized as key drivers of UC pathogenesis (Haberman et al., 2019; Madill-Thomsen et al., 2025). Conversely, downregulated genes were significantly enriched in fatty acid oxidation and mitochondrial electron transport/respiration pathways, including CPT1A, PPARα. This suggests a metabolic shift from oxidative phosphorylation toward glycolysis, in line with immunometabolic reprogramming in proinflammatory macrophages (Kelly and O'Neill, 2015; Liu et al., 2021; Wu et al., 2025). Importantly, this observation aligns with the scRNA-seq findings, reinforcing the reproducibility of metabolic alterations across data modalities.

A major contribution of this study is the identification of macrophage-associated immunometabolic signatures linked to UC through the integration of scRNA-seq, bulk transcriptomics, WGCNA, and machine-learning approaches. By integrating WGCNA with three complementary machine learning algorithms, we identified six core metabolic regulators: CYBB, CR1, INPP5D, CTSH, IFI16, and NCF4. CYBB encodes NADPH oxidase, promoting ROS generation and sustaining M1 proinflammatory polarization (Jeong et al., 2023). CR1 (CD35) is a complement receptor that regulates complement activation by binding C3b/C4b and inhibiting C3/C5 convertases; alterations in CR1 expression or activity under chronic inflammation may amplify complement-driven signaling and thereby modulate the intensity of inflammatory responses (Khera and Das, 2009). INPP5D (encoding SHIP-1) hydrolyzes PI(3,4,5)P_3_ to PI(3,4)P_2_, thus suppressing downstream PI3K-Akt signaling, restricts survival and metabolic activation signals in myeloid/macrophage populations, influencing cell fate and metabolic reprogramming under inflammatory conditions (Yeoh and Krebs, 2023). NCF4, a regulatory subunit of the NADPH oxidase complex (p40^phox), contributes to ROS generation during inflammatory activation and partially maintains proinflammatory macrophage polarization (Li et al., 2024). CTSH, a lysosomal cysteine protease, is crucial for protein degradation and lysosomal function. Altered expression or activity of CTSH can affect inflammatory regulation (Menzel et al., 2006). IFI16, a PYHIN family member, acts as a DNA sensor. It recognizes aberrant or pathogenic DNA, then triggers downstream interferon and NF- κB signaling, which promotes cytokine secretion during inflammation (Li et al., 2024). While CYBB and IFI16 have been extensively linked to inflammation, evidence directly connecting CR1, NCF4, and CTSH to UC remains limited. Although CYBB and IFI16 have been extensively linked to inflammatory signaling, the roles of CR1, NCF4, and CTSH in UC remain less well characterized. Notably, previous work by Menzel et al. demonstrated altered cathepsin activity in IBD macrophages. Therefore, our findings should be considered supportive and confirmatory rather than the first evidence implicating CTSH in intestinal inflammation.

To further substantiate these computational findings, we performed histological and protein-level validation using colonic tissues from UC patients and healthy controls. H&E staining revealed typical pathological features of UC, including crypt distortion, epithelial disruption, goblet cell depletion, and dense inflammatory infiltration in the lamina propria, confirming the presence of active mucosal inflammation. Consistent with these observations, Western blot analysis demonstrated significant upregulation of the six macrophage-associated metabolic regulators- CR1, INPP5D, CYBB, IFI16, NCF4, and CTSH - in UC tissues. Densitometric quantification showed respective fold increases of 1.80, 2.87, 2.02, 1.58, 1.66, and 1.53, closely aligning with the transcriptomic predictions. These histological and molecular results provide direct biological evidence supporting macrophage-associated metabolic activation in UC and bridge computational inference with *in vivo* validation. Collectively, they reinforce the concept that macrophage immunometabolic reprogramming represents both a molecular signature and a histopathological hallmark of UC mucosal inflammation.

Correlation analysis revealed CYBB and CR1 as the strongest markers of macrophage infiltration, while GSEA confirmed their enrichment in complement and cytokine–receptor interaction pathways, reinforcing their immunometabolic roles. Consensus clustering of 148 UC samples further stratified patients into two molecular subtypes: Cluster 1, with elevated expression of CYBB, CTSH, and CR1, exhibited a metabolically active, proinflammatory phenotype. ROC analyses demonstrated that all six feature genes achieved AUC values >0.98 in validation cohorts, with IFI16 and CYBB showing the highest predictive performance. Collectively, these findings highlight metabolic reprogramming as a key determinant of UC heterogeneity, supporting gene-based stratification as a basis for personalized management.

Chronic inflammation–induced metabolic remodeling amplifies ROS generation, disrupts lipid and energy metabolism, and contributes to tissue damage and fibrosis (Kelly and O'Neill, 2015; Garrido-Trigo et al., 2023). Notably, Recent studies have begun to explore these processes: Hong et al. demonstrated macrophage metabolic dysfunction in a DSS-induced UC model, with upregulation of NOX2 complex subunits including CYBB (Hong et al., 2024), while Garrido-Trigo et al. uncovered pronounced heterogeneity of intestinal macrophages at single-cell resolution in human inflamed colon (Garrido-Trigo et al., 2023). However, these studies mainly described broad cellular or metabolic alterations without pinpointing specific regulators or evaluating diagnostic potential.

Notably, several of these genes (CYBB and NCF4) are components of the NADPH oxidase complex, implicating reactive oxygen species (ROS) generation as a central feature of macrophage metabolic activation. Additionally, INPP5D links these processes to PI3K-Akt signaling, suggesting coordinated regulation of inflammatory and metabolic pathways.

Importantly, the screening strategy incorporated scRNA-seq-derived macrophage marker genes. These genes primarily identify macrophage cell identity and do not necessarily represent causal regulators of macrophage function. Therefore, the six genes identified in this study should be interpreted as macrophage-associated immunometabolic markers that correlate with macrophage abundance and inflammatory activity rather than definitive drivers of macrophage metabolic reprogramming. Additional mechanistic studies, including macrophage-specific perturbation experiments, will be required to establish causality.

The high AUC values observed in both training and validation cohorts warrant careful interpretation. Although multiple machine-learning algorithms converged on the same feature set and independent GEO cohorts were used for validation, the relatively limited sample size and repeated feature-selection procedures increase the possibility of optimistic performance estimates. Future studies should evaluate these biomarkers in larger multicenter cohorts using rigorous external validation and nested cross-validation frameworks.

### 4.1 Limitations

This study has several limitations. First, the scRNA-seq dataset included only UC samples and lacked healthy controls, limiting identification of UC-specific macrophage alterations. Second, the identified genes should be interpreted as candidate macrophage-associated immunometabolic markers rather than definitive metabolic regulators, as macrophage marker genes primarily reflect cellular identity. Third, the experimental validation cohort was small (n = 3 per group) and provides only preliminary biological confirmation. Fourth, despite independent external validation, potential overfitting cannot be excluded because of the multi-step feature-selection process and modest sample size. Finally, as an observational transcriptomic study, it cannot establish causal relationships without further functional validation.

## 5 Conclusion

Our multi-omics analysis identified six candidate macrophage-associated immunometabolic markers (CYBB, CR1, INPP5D, CTSH, IFI16, and NCF4) with potential mechanistic relevance and diagnostic value in UC. Combined with histological and protein-level validation, these findings broaden the understanding of macrophage immunometabolism and reveal potential biomarkers for disease stratification. Importantly, our results emphasize macrophage metabolic reprogramming as a central driver of UC inflammation and provide a foundation for future investigation into precision-based therapeutic strategies.

## Data Availability

The data presented in this study are deposited in the GEO repository (https://www.ncbi.nlm.nih.gov/geo/), accession numbers GSE162335, GSE48958, GSE73661, GSE75214, and GSE16879.
