# Supplementary material for: Multi-omics analysis identifies macrophage immunometabolic signatures in ulcerative colitis
Source: Front Cell Infect Microbiol. 2026 Sep 3;16:1858403. doi: 10.3389/fcimb.2026.1858403 (PMC13581638; doi:10.3389/fcimb.2026.1858403)
Supplement: Supplementary file 1 [file Table1.docx]

Supplementary Table 1 Information of NCBI GEO Datasets

|  |  | **Accession ID** | **Type** | **Tissue** | **Disease** | **Control** | **Platform** |
| --- | --- | --- | --- | --- | --- | --- | --- |
| ulcerative colitis） | Analysis Dataset | GSE162335 | scRNA-seq | Colon | 11 | 0 | GPL20301 Illumina HiSeq 4000 (Homo sapiens) |
|  |  | GSE48958 | RNA-seq | Colon | 7 | 8 | GPL6244 [HuGene-1_0-st] Affymetrix Human Gene 1.0 ST Array [transcript (gene) version] |
|  |  | GSE73661 | RNA-seq | Colon | 67 | 12 | GPL6244 [HuGene-1_0-st] Affymetrix Human Gene 1.0 ST Array [transcript (gene) version] |
|  |  | GSE75214 | RNA-seq | Colon | 74 | 11 | GPL6244 [HuGene-1_0-st] Affymetrix Human Gene 1.0 ST Array [transcript (gene) version] |
|  | Validation Dataset | GSE16879 | RNA-seq | Colon | 30 | 0 | GPL570 [HG-U133_Plus_2] Affymetrix Human Genome U133 Plus 2.0 Array |
