# Supplementary material for: Multi-omics analysis identifies macrophage immunometabolic signatures in ulcerative colitis
Source: Front Cell Infect Microbiol. 2026 Sep 3;16:1858403. doi: 10.3389/fcimb.2026.1858403 (PMC13581638; doi:10.3389/fcimb.2026.1858403)
Supplement: Supplementary file 2 [file Table3.docx]

Here is a link for only Table 5:

<https://fromsmash.com/T6aNZJqrVr-dt>
